# Supplementary material for: Epilepsy and nodding syndrome in association with an Onchocerca volvulus infection drive distinct immune profile patterns
Source: PLoS Negl Trop Dis. 2023 Aug 3;17(8):e0011503. doi: 10.1371/journal.pntd.0011503 (PMC10426931; doi:10.1371/journal.pntd.0011503)
Supplement: S1 STROBE Checklist — (DOC) [file pntd.0011503.s001.doc]

STROBE Statement—Checklist of items that should be included in reports of ***cross-sectional studies***

|  | Item No | Recommendation |
| --- | --- | --- |
| **Title and abstract** | 1 | (*a*) Indicate the study’s design with a commonly used term in the title or the abstract  **This study evaluated different parameters (immunological, neurological and worm-related metabolic product) in individuals (age range 7-54 years old) from the Mahenge area, Tanzania, presenting with either epilepsy or nodding syndrome with or without *Onchocerca volvulus* infection and compared them to *O. volvulus* negative individuals from the same endemic area lacking neurological disorders.** |
|  |  | (*b*) Provide in the abstract an informative and balanced summary of what was done and what was found  **The association of onchocerciasis (caused by *O. volvulus*) with epilepsy, including nodding syndrome is known for many years although the pathophysiological mechanisms are unsolved so far. The present study, performed in the Mahenge area, in south eastern Tanzania between November 2014 and April 2015, investigated the immune profile of individuals who were displaying epilepsy/nodding syndrome in absence or presence of a PCR-determined *O. volvulus* infection. The group of *O. volvulus* infected patients was characterized by higher levels of eosinophils, antigen-specific immunoglobulin levels as well as increased levels of a worm-related metabolic product (NATOG), which might be an interesting diagnostic biomarker candidate in future studies** |
| Introduction | | |
| Background/rationale | 2 | Explain the scientific background and rationale for the investigation being reported  **This study aimed at identifying clinical features and potential etiology of epilepsy/nodding syndrome in the Mahenge area, Ulanga district, Tanzania.** |
| Objectives | 3 | State specific objectives, including any prespecified hypotheses  **Pages 5-6.** |
| Methods | | |
| Study design | 4 | Present key elements of study design early in the paper  **Pages 5-6.** |
| Setting | 5 | Describe the setting, locations, and relevant dates, including periods of recruitment, exposure, follow-up, and data collection  **Pages 7-8.** |
| Participants | 6 | *(*a) Give the eligibility criteria, and the sources and methods of selection of participants  **Pages 7-8.** |
| Variables | 7 | Clearly define all outcomes, exposures, predictors, potential confounders, and effect modifiers. Give diagnostic criteria, if applicable  **Pages 7-8.** |
| Data sources/ measurement | 8* | For each variable of interest, give sources of data and details of methods of assessment (measurement). Describe comparability of assessment methods if there is more than one group  **Page 7-11 (Methods).** |
| Bias | 9 | Describe any efforts to address potential sources of bias  **n/a.** |
| Study size | 10 | Explain how the study size was arrived at  **Between November 2014 and April 2015, 187 individuals from the Mahenge area, Ulanga district, in south eastern Tanzania, participated in this survey. Of those 187 individuals, 144 individuals provided blood samples (as indicated in Figure S1) and out of them, 106 presented neurological symptoms determined by a neuropaediatrician and a neurology resident. We assessed a further 38 individuals without neurological signs/symptoms that were age, gender and village matched. Table 1 shows details about the different groups within the study cohort.** |
| Quantitative variables | 11 | Explain how quantitative variables were handled in the analyses. If applicable, describe which groupings were chosen and why  **Statistical tests to compare different parameters between the indicated groups were performed on Prism software (GraphPad Prism, version 9.4.1). Before testing for statistical significances between the groups, we performed a D’Agostino-Person omnibus normality test to test the distribution of the values. Kruskal Wallis followed by Dunn’s multiple comparisons tests were used to compare control individuals and people with epilepsy and those with nodding syndrome with or without an *O. volvulus* infection determined by PCR. For comparisons of continuous parameters, the Spearman correlation test was used. An odds ratio (OR) was calculated with a 95% confidence interval using SPSS software version 27.0 (IBM, New York, USA). P-values of ≤ 0.05 were considered significant.** |
| Statistical methods | 12 | (*a*) Describe all statistical methods, including those used to control for confounding  **Page 10-11.** |
|  |  | (*b*) Describe any methods used to examine subgroups and interactions  **Page 10-11.** |
|  |  | (*c*) Explain how missing data were addressed  **As indicated in Figure S1, out of 144 individuals, blood smears were available from 79 participants, 61 provided urine and from 103 individuals plasma samples were usable.** |
|  |  | (*d*) If applicable, describe analytical methods taking account of sampling strategy  **Page 7-8.** |
|  |  | (*e*) Describe any sensitivity analyses  **n/a.** |
| Results | | |
| Participants | 13* | (a) Report numbers of individuals at each stage of study—eg numbers potentially eligible, examined for eligibility, confirmed eligible, included in the study, completing follow-up, and analysed  **Page 7-8, Figure S1.** |
|  |  | (b) Give reasons for non-participation at each stage  **n/a.** |
|  |  | (c) Consider use of a flow diagram  **Figure S1.** |
| Descriptive data | 14* | (a) Give characteristics of study participants (eg demographic, clinical, social) and information on exposures and potential confounders  **Page 7-8, Table 1.** |
|  |  | (b) Indicate number of participants with missing data for each variable of interest  **Figure S1.** |
| Outcome data | 15* | Report numbers of outcome events or summary measures  **Page 11-15.** |
| Main results | 16 | (*a*) Give unadjusted estimates and, if applicable, confounder-adjusted estimates and their precision (eg, 95% confidence interval). Make clear which confounders were adjusted for and why they were included  **Provided in individual figure legends for each data set.** |
|  |  | (*b*) Report category boundaries when continuous variables were categorized  **See results.** |
|  |  | (*c*) If relevant, consider translating estimates of relative risk into absolute risk for a meaningful time period  **n/a.** |
| Other analyses | 17 | Report other analyses done—eg analyses of subgroups and interactions, and sensitivity analyses  **Page 15.** |
| Discussion | | |
| Key results | 18 | Summarise key results with reference to study objectives  **Page 16-21.** |
| Limitations | 19 | Discuss limitations of the study, taking into account sources of potential bias or imprecision. Discuss both direction and magnitude of any potential bias  **Page 20-21.** |
| Interpretation | 20 | Give a cautious overall interpretation of results considering objectives, limitations, multiplicity of analyses, results from similar studies, and other relevant evidence  **Page 20-21.** |
| Generalisability | 21 | Discuss the generalisability (external validity) of the study results  **See abstract.** |
| Other information | | |
| Funding | 22 | Give the source of funding and the role of the funders for the present study and, if applicable, for the original study on which the present article is based  **Deutsche Gesellschaft für Epilepsie, German Center of Infectious Disease, BMBF, Deutsche Forschungsgemeinschaft. This is an original study; the funders had no role in the study.** |

*Give information separately for exposed and unexposed groups.

**Note:** An Explanation and Elaboration article discusses each checklist item and gives methodological background and published examples of transparent reporting. The STROBE checklist is best used in conjunction with this article (freely available on the Web sites of PLoS Medicine at http://www.plosmedicine.org/, Annals of Internal Medicine at http://www.annals.org/, and Epidemiology at http://www.epidem.com/). Information on the STROBE Initiative is available at www.strobe-statement.org.
