# Supplementary material for: Epilepsy and nodding syndrome in association with an Onchocerca volvulus infection drive distinct immune profile patterns
Source: PLoS Negl Trop Dis. 2023 Aug 3;17(8):e0011503. doi: 10.1371/journal.pntd.0011503 (PMC10426931; doi:10.1371/journal.pntd.0011503)
Supplement: S2 Table — (DOCX) [file pntd.0011503.s003.docx]

**S2 Table. Chemokines and neurodegeneration markers.**

|  | **CTRL (n=17)** | **EpNd (n=32)** | **OV^+^ EpNd (n=54)** | **p value** |
| --- | --- | --- | --- | --- |
| **IL-8 pg/ml (median and IQR)** | 10.72 (6.70, 34.83) | 11.36 (7.30, 14.33) | 12.21 (5.82, 18.9) | 0.828 |
| **IP-10 pg/ml (median and IQR)** | 709 (237.1, 1149) | 583.6 (346.2, 894.7) | 326.5 (199.4, 694.1) | 0.055 |
| **MIP-1β pg/ml (median and IQR)** | 167.4 (154, 266.8) | 232.8 (175.4, 287.2) | 196.6 (159.8, 271.9) | 0.098 |
| **RANTES MFI (median and IQR)*** | 6686 (5472, 7152) | 6542 (5813, 7701) | 6529 (5694, 7645) | 0.829 |
| **Amyloid-β 1-40 pg/ml (median and IQR)** | 9.23 (9.23, 9.23) | 9.23 (9.23, 9.23) | 9.23 (9.23, 9.23) | 0.090 |
| **FGF-21 pg/ml (median and IQR)** | 8.64 (1.48, 112.7) | 36.32 (8.64, 106.4) | 77.1 (8.64, 296.9) | 0.051 |
| **Kallikrein-6 pg/ml (median and IQR)** | 1402 (1121, 2077) | 1513 (1042, 2080) | 1445 (1044, 2093) | 0.921 |
| **NCAM-1 pg/ml (median and IQR)** | 23674 (54, 49532) | 23031 (5502, 88583) | 20727 (5929, 113536) | 0.450 |
| **Tau total pg/ml (median and IQR)** | 20 (6.94, 24.53) | 20 (18.81, 20) | 20 (6.94, 22.27) | 0.050 |
| **Tau pT181 pg/ml (median and IQR)** | 1.95 (1.95, 1.95) | 1.95 (1.95, 1.95) | 1.95 (1.95, 1.95) | 0.741 |

Levels of plasma-derived chemokines and neurodegeneration markers were determined using Luminex technology; data were tested for significance using Kruskal Wallis tests. IQR: interquartile ranges, * RANTES values are given as mean fluorescence intensity (MFI) instead of concentrations due to improper standard curve.
