# Supplementary material for: Epilepsy and nodding syndrome in association with an Onchocerca volvulus infection drive distinct immune profile patterns
Source: PLoS Negl Trop Dis. 2023 Aug 3;17(8):e0011503. doi: 10.1371/journal.pntd.0011503 (PMC10426931; doi:10.1371/journal.pntd.0011503)
Supplement: S3 Table — MF load was correlated with the optical density of O. volvulus-specific immunoglobulins determined by ELISA from all available plasma samples (n = 99). A Spearman correlation test was applied. (DOCX) [file pntd.0011503.s004.docx]

**S3 Table. Correlations of microfilarial load and O. volvulus-specific Igs.**

|  | **Spearman rho** | **p-value** |
| --- | --- | --- |
| ***O. volvulus*-specific IgG1** | 0.298 | 0.003 |
| ***O. volvulus*-specific IgG2** | 0.317 | 0.001 |
| ***O. volvulus*-specific IgG3** | 0.305 | 0.002 |
| ***O. volvulus*-specific IgG4** | 0.284 | 0.004 |
| ***O. volvulus*-specific IgE** | 0.330 | 0.001 |

MF/mg were correlated to the levels of O. volvulus-specific Igs. p values denote statistical differences for these parameters tested by Spearman correlation test.
