# Supplementary figures and images for: Epilepsy and nodding syndrome in association with an Onchocerca volvulus infection drive distinct immune profile patterns
Source: PLoS Negl Trop Dis. 2023 Aug 3;17(8):e0011503. doi: 10.1371/journal.pntd.0011503 (PMC10426931; doi:10.1371/journal.pntd.0011503)

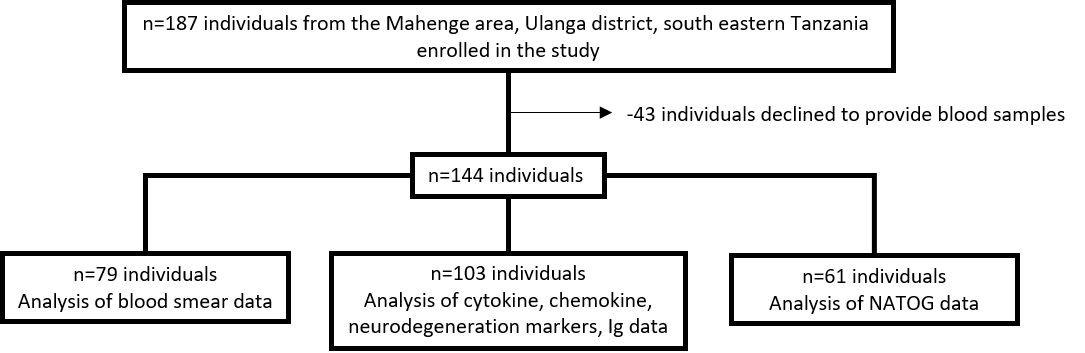

Supplement: S1 Fig — (TIF) [file pntd.0011503.s006.tif]

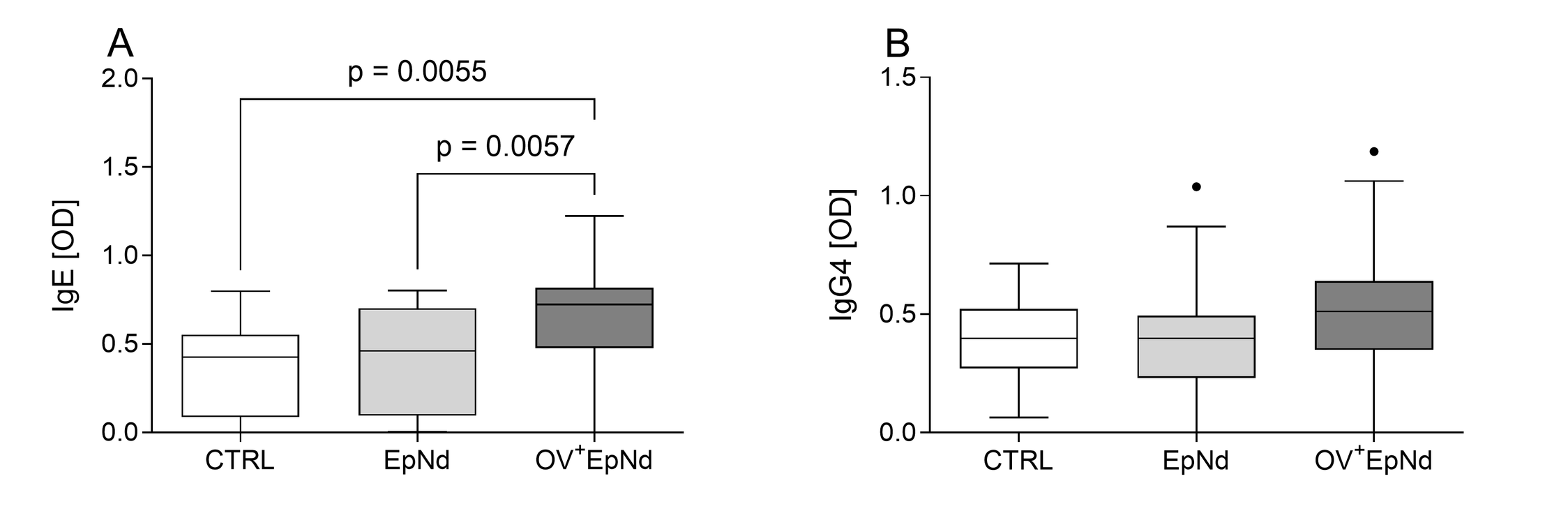

Supplement: S2 Fig — Levels of total (A) IgE and (B) total IgG4 from plasma samples (CTRL, n = 16, EpNd, n = 32, OV+EpNd, n = 51) were measured using ELISA technology. Graphs show box whiskers with median, interquartile ranges and outliers. Statistical significances between the indicated groups were obtained with Kruskal-Wallis followed by a Dunn’s multiple comparison test. (TIF) [file pntd.0011503.s007.tif]
