# Supplementary material for: Epilepsy and nodding syndrome in association with an Onchocerca volvulus infection drive distinct immune profile patterns
Source: PLoS Negl Trop Dis. 2023 Aug 3;17(8):e0011503. doi: 10.1371/journal.pntd.0011503 (PMC10426931; doi:10.1371/journal.pntd.0011503)
Supplement: Attachment — Submitted filename: Reviewers comments pbp 20230530.docx [file pntd.0011503.s008.docx]

**Reviewer 1**

**We thank the reviewer for taking the time to thoroughly read our manuscript and are pleased that they consider the work of interest. We have addressed all the issues that were raised, and our answers are detailed in the section below. All changes in the manuscript have been highlighted in yellow.**

**Methods**

Reviewer #1: Major revisions:

- Line 171: you state in the methodology section that the cases and controls were matched by age and sex. But then you never mention or describe this matching. For example, what is the accepted age range for matching?

**Thank you for picking up this aspect. To be precise, patients were matched by ancestry and origin, not by age and gender. Therefore, we change the paragraph as follows:**

***Furthermore, uninfected controls from the same villages from which the patients came were included (lines 164-165).***

**However, as can be seen in the results section, age and gender distribution was comparable between the three groups (Table 1).**

And how did you match 38 controls and 144 cases?

**We apologize for the misleading wording and rephrase the text as follows:**

***In total, we had 187 individuals that initially participated in the study and provided skin snips for the performance of the* O. volvulus-*specific PCR. Out of these 187 individuals, only 144 provided blood samples and they consist of 106 cases that suffered from Epilepsy/Nodding syndrome composed of 61 individuals with* O. volvulus *infection (OV^+^EpNd) and 45 individuals without* O. volvulus *infection (EpNd). Additionally 38 controls who were O. volvulus PCR negative were included as depicted in Table 1 (lines 247-252).***

Minor revisions:

- The ethics section might be more relevant at the end of the methodology section. We are better able to assess ethical considerations after having a clear description of the methodology.

**We thank the reviewer for this suggestion and have adapted the material and method section accordingly; thus, the ethics section is now moved at the end of this part of the paper (lines 236-243).**

- Line 142: meaning of regular care? Please specify "regular care" in the study area.

**We thank the reviewer for pointing out this inaccurate wording and changed the description to the following:**

***The majority of the included individuals with epilepsy/nodding syndrome were already known patients at the Mahenge Epilepsy Clinic or at surrounding public health facilities where they received their anti-seizure medications monthly (lines 134-136).***

- Line 149: including routine interictal EEG in most cases. Please specify "most cases".

**We changed it to the following wording:**

***… in all cases that were willing to have one performed (95/106, lines 142-143).***

**Of note: not all individuals received an EEG because some individuals had no time anymore to wait (some individuals would have had to wait one hour or more to receive the EEG, so they stopped. However, these were only few isolated cases).**

- Line 152: Standardized and validated questionnaire. How was it validated?

**Here, we rephrased it to simply “*a standardized questionnaire*” (line 146).**

- Line 157: concomitant cardiovascular and renal disease were excluded. Why?

**Based on the ethical approved study plan subjects with cardiovascular and renal disease were excluded, since these diseases and especially medication influence humoral and cellular immune responses, cell composition in the blood, and haematological and urine parameters.**

- No sample calculation. Why?

**Indeed, this is an important point, as a prior power calculation would statistically underpin the results obtained. However, the number is based on the previous description of Nodding syndrome in Tanzania by our research group (Winkler et al. 2008), as we wanted to include a comparable and representative number of patients with nodding syndrome and epilepsy. Since, to our knowledge, no cohort of nodding syndrome patients larger than 63 has been described so far (Winkler et al. 2008), and also considering the exploratory nature of the study, the number of about 60 cases per study group (OV^+^ EpNd, EpNd, controls) seemed appropriate to us (187 individuals were recruited of which 144 patients’ biomaterial was available (Supplementary Figure 1).**

**Results**

Reviewer #1: Major revisions:

- The representativeness of the sample was not easy to verify. A table describing the two groups with more variables other than sex and age might help us better appreciate the description of individuals, and potential confounding. The evolution/variation of immune cells and chemokines can be influenced by many reasons that we cannot attempt to appreciate even a little here.

**We thank the reviewer for this suggestion and included some additional information about the participants in a Supplementary Table 1, and amended in the results section the following statement:**

***The overall median of age at investigation was 22 years [range 7-54 years] and the median of age at onset of nodding syndrome or epilepsy was 9 years [range 2-46 years]. Most individuals worked as farmers (98/144) or still went to school (12/144). Information on comorbidities, current anti-seizure medication and history of ivermectin intake in the past five years (via WHO African Programme for Onchocerciasis Control)(42) are listed in Supplementary Table 1 (lines 258-262).***

**Summary and General Comments**

Reviewer #1: The authors present a study to investigate immunological parameters in individuals from Tanzania with epilepsy or nodding syndrome with or without O. volvulus infection and compared to negative individuals from the same endemic area without neurological disorders.

This study was original, and gives relevant data in the field of epilepsy and/or Nodding syndrome and the association with immunological disturbances. The manuscript is well written and easy to read, except for the discussion section which is a bit long and dilutes the key messages.

**We thank the reviewer for that suggestion and amended/shortened the discussion part accordingly (lines 358-492).**
